# Ethyl Chlorid Anesthesia by the Open Method

**Published:** 1911-03-15

**Authors:** C. Edson Abbott

**Affiliations:** Franklin, Mass.


### ETHYL CHLORID ANESTHESIA BY THE OPEN METHOD.

BY C. EDSON ABBOTT, D.D.S., FRANKLIN, MASS.

A short, safe, agreeable and convenient general anesthesia is needed for the extraction of teeth, short dental surgical operations, lancing abscesses, etc. If needed extractions are referred to a specialist who does them well under proper aseptic conditions, well and good; too often, however, extractions imperatively demanded for the health of the patient are omitted because the dentist has no sufficient and simple anesthesia.

*Method of Administration.* The patient is seated in the chair, with clothing loosend: the mouth is thoroughly cleansed and assurances are given that the operation will be safe, painless and soon over. The skilled operator can safely guarantee this in every case. A six by six aseptic napkin, folded to make four thicknesses, is held over the mouth, which is held wide open by a suitable rubber gag, which the experienced operator will sometimes dispense with. The writer then sprays ethyl chlorid from a 100-gram metal tube on to the center of the gauze and instructs the patient to breathe deeply and fully. The induction should take forty seconds. The full anesthesia lasts ninety seconds, and the recovery to the usual feelings will take only a few moments. I find that this cloth has the advantage over the usual inhaler in being absolutely clean, not productive of fright in timid patients, absolute certainty of plenty of air, simplicity and convenient, particularly in cases of fright and struggling by children or alcoholics. The writer tests the completeness of anesthesia by lifting the patients' arm, which should be limp. Usually the patient goes to sleep like a baby and wakes up smiling. Occasionally screaming, vomiting and some struggling occur, caused by the swallowing of blood, previous fear or excitement, fatigue caused by previous pain, unusual physical conditions, etc. It is believed that after dentists master the technic they will, like the author, find this superior to all other methods of short anesthesia in absolute certainty, simplicity, short induction, long available anesthesia, fewer after-effects, least expense, safety and portability. Many operators avoid general anesthesia because the conditions have such complexity and paraphernalia. The method above described can be used by every careful operator in his everyday work.—*Dental Brief.*

